# Images in Black and White: Disparities in Utilization of Computed Tomography and Ultrasound for Older Adults with Abdominal Pain

**DOI:** 10.5811/westjem.18087

**Published:** 2025-02-28

**Authors:** Ijeoma C. Unachukwu, Michael N. Adjei-Poku, Olivia C. Sailors, Rachel Gonzales, Eugenia South, Zach Meisel, Rachel R. Kelz, Anne R. Cappola, Ari B. Friedman

**Affiliations:** * University of Pennsylvania, Department of Emergency Medicine, Philadelphia, Pennsylvania; † University of Pittsburgh Medical Center, Department of Psychiatry, Pittsburgh, Pennsylvania; ‡ University of Pennsylvania, Leonard Davis Institute of Health Economics, Philadelphia, Pennsylvania; § University of Pennsylvania, Urban Health Lab, Philadelphia, Pennsylvania; ¶ University of Pennsylvania School of Medicine, Institute of Aging, Philadelphia, Pennsylvania; || University of Pennsylvania, Center for Surgery and Health Economics, Philadelphia, Pennsylvania; # University of Pennsylvania, Department of Endocrinology, Philadelphia, Pennsylvania

## Abstract

**Introduction:**

Abdominal pain is the leading emergency department (ED) chief complaint in older (≥65 years of age) adults, accounting for 1.4 million ED visits annually. Ultrasound and computed tomography (CT) are high-yield tests that offer rapid and accurate diagnosis for the most clinically significant causes of abdominal pain. In this study we used nationally representative data to examine racial/ethnic differences in cross-sectional imaging for older adults presenting to the ED with abdominal pain.

**Methods:**

We performed a retrospective, cross-sectional analysis using data from the National Hospital Ambulatory Medical Care Survey (NHAMCS) to assess differences in the rate of imaging between White and Black older adults presenting to the ED for abdominal pain. Our primary outcome was the receipt of abdominal CT and/or ultrasound imaging.

**Results:**

Across 1,656 older adult ED visits for abdominal pain, White patients were 26.8% (relatively, 14.2% absolute) more likely to receive abdominal CT and/or ultrasound than Black patients: 802 of 1,197 (67.0%) White patients were 26.8% (relatively, 14.2% absolute) more likely to receive abdominal computed tomography and/ or ultrasound than Black patients (P=0.01).

**Conclusion:**

This study revealed that Black older adults presenting to the ED with abdominal pain receive significantly lower levels of cross-sectional imaging (CT/ultrasound) than White patients. Our findings highlight the need for further investigations into causes of disparities while initiating quality improvement processes to assess and address site- and clinician-specific patterns of care.

## INTRODUCTION

Abdominal pain is the leading emergency department (ED) chief complaint in older (≥65) adults, accounting for 1.4 million ED visits annually.^1^ Abdominal pain in older adults has a broad differential diagnosis and morbidity and mortality equal to or greater than that of ST-elevation myocardial infarction.^2^ Timely diagnosis and treatment are crucial for optimal outcomes. Although questions remain about the optimal level of testing, ultrasound and computed tomography (CT) (collectively referred to as cross-sectional imaging) offer rapid, accurate diagnosis of most clinically significant, treatable causes of abdominal pain.^3^,^4^ However, 40% of older adults with abdominal pain receive neither an abdominal ultrasound nor CT in EDs nationally.^5^

There are no objective signs, symptoms, standards or lab tests that can perfectly determine the need for CT.^6^ In this context, individual clinical judgment and local practice patterns can determine which patients receive these tests and may engender disparate care between Black and White patients.^6^,^7^ Prior research on the national scale provides adequate evidence suggesting minoritized populations receive significantly less imaging overall compared to their White counterparts.^8^,^9^ Given known disparities in the assessment and treatment of pain for Black patients, there may be disparities in imaging based on a chief complaint of abdominal pain because there are no guidelines for the management of acute, geriatric abdominal pain.^6^,^10^,^11^ A cohort of ED patients at a single institution provides suggestive evidence of such an effect: Black and Hispanic adults (≥18) with abdominal pain were significantly less likely to receive CT than their White counterparts.^7^

There is currently a paucity of studies assessing the racial disparities in imaging in abdominal pain in older adults. In this study we used nationally representative data to examine patterns of cross-sectional imaging for abdominal pain presentations among older adults in US EDs. We hypothesized that non-Hispanic Black (Black) patients with a chief complaint of abdominal pain would receive cross-sectional (CT and/or ultrasound) imaging at lower rates than non-Hispanic White (White) patients.

## METHODS

### Study Setting and Population

To test this hypothesis, we performed a retrospective, cross-sectional analysis using data from the National Hospital Ambulatory Medical Care Survey (NHAMCS) to assess for differences in the rate of imaging between White, Black, and Hispanic older adults presenting to the ED for abdominal pain. We pooled data from 2013–2020. The NHAMCS is an annual, cross-sectional survey conducted by the National Center for Health Statistics (NCHS) of the US Centers for Disease Control and Prevention, which abstracts ED charts from non-federal, acute care hospitals.^12^ The survey design is fully described elsewhere.^12^

We restricted our sample to older adults (≥65 years old) with an abdominal pain chief complaint. The NHAMCS uses pre-established reason-for-visit classification schemes to encode the free text recorded in the chart into one of 5,449 standardized chief complaints.^12^ We defined a chief complaint of abdominal pain as the patient’s report of pain-like symptoms of either the abdomen or of any abdominal internal organ as the primary reason for visit. Most diagnoses came from the code “Abdominal pain, cramps, or spasms not otherwise specified.” Additional methodology for selecting abdominal pain chief complaints in this dataset have been previously described.^5^ (Supplemental Table 1)

**Population Health Research Capsule**

What do we already know about this issue?

*The decision to image older adults with abdominal pain is high stakes yet lacks guidelines. Reliance on clinical judgment could result in bias.*

What was the research question?

*Are there significant racial differences in ultrasound and computed tomography use in older adults presenting to the ED with abdominal pain?*

What was the major finding of the study?

*White patients were 26.8% more likely to receive abdominal computed tomography and/or ultrasound than Black patients (P=0.01).*

How does this improve population health?

*Understanding the presence of disparities in vulnerable populations is essential to rectifying biased cognitive patterns in patient care.*

### Outcomes and Primary Comparison

Our primary, dichotomous outcome was the receipt of abdominal CT, ultrasound, or combined CT and/or ultrasound imaging among older adults presenting with abdominal pain during their visit. We excluded magnetic resonance imaging as it was not a standard for rapid diagnosis and was used in <5 ED visits.

### Independent Variables

Independent variables included racial and ethnic groups (hereby defined as race/ethnicity), sociodemographics, and hospital characteristics. We categorized patients according to NHAMCS racial and ethnic categories as abstracted from ED charts: non-Hispanic White; non-Hispanic Black; and Hispanic. We dropped American Indian and Alaska Native groups due to insufficient sample size (<30). The primary comparison was between Black and White patients for two reasons. First, prior studies have demonstrated that the widest and most consistent disparities across a variety of health outcomes exist between Black and White patients, one being a minority group subject to both structural and individual racism, and the other a historically privileged majority.^13^,^14^ Second, power analyses between all racial/ethnic groups suggested the strongest power between these groups regarding combined CT/ultrasound imaging (>80% power at alpha=0.05). Similar power analyses between Hispanic and White patients did not yield adequate power (<60%).

### Covariates

For each patient, we analyzed sociodemographic information: age; biological sex (gender not recorded in data); residence; insurance; and triage level. Age categories were designated as 65–74, 75–84, and ≥85 years. Residence categories included the following: homeless; nursing home; living at home; or living in a private institution. Insurance categories were Medicaid, Medicare, private insurance, self-pay, and unspecified. Triage levels were defined as Emergency Severity Index (ESI): ESI 1, immediate; ESI 2, emergent; ESI 3, urgent; ESI 4, semi-urgent; and ESI 5, non-urgent. We excluded ESI 1 from analysis primarily because this category had an insufficient sample size in the preliminary data (<30). In addition, prior literature has demonstrated that abdominal pain is a rare chief complaint of ESI 1 or similar high-acuity category presentations.^13^,^15^,^16^ Research indicates that the highest acuity patients frequently either lack the time or capability to reliably report their chief complaint as they require acute and immediate care.^5^ The NCHS re-scales EDs that use a different triage system to the 5-level ESI scale using a validated methodology.^12^

### Statistical Analysis

We used standard descriptive statistics (counts and percentages of binary and categorical variables) to report the characteristics of each visit and chi-square tests to compare study variables across racial/ethnic groups, computed with Stata version 15.1 (StataCorp, College Station, TX). Individual chi-square tests were run per imaging modality. All statistical analyses and estimates were done using NHAMCS four-level, probability-based survey weights to estimate nationally representative statistics, including 95% confidence intervals and hypothesis tests. These weights were adjusted for non-response by time of year, geographic region, urbanicity, and hospital ownership. All hypothesis tests were two-sided with alpha = 0.05. We omitted or combined cells with fewer than 30 observations. Missingness was <2% and addressed with row-wise deletion. The Penn Institutional Review Board exempted this de-identified analysis from review.

## RESULTS

### Patient and Hospital Characteristics

Our sample included 1,656 abdominal pain ED visits from older adults from 2013–2020 (Table). Based on survey weights, these sampled visits represent an estimated eight-year incidence of 12,553,136 older adult abdominal pain ED visits. Visits from White patients comprised much of the sample (1,197, or 72.3% after applying survey weights). Black (234, 14.1%) and Hispanic (153, 9.2%) patient visits were also common (Table 1). Across all races and ethnicities, the average patient was 76.1 years old. The majority of visits were from patients who were women (1,013, 61.2%), those living in their own homes (1,529, 92.4%), those living in urban areas (1,373, 82.9%), and patients with Medicare insurance (1,322, 79.8%).

### Administration of Cross-sectional Imaging

Across all older adult (≥65 years of age) ED visits for abdominal pain, cross-sectional imaging (CT and/or ultrasound) was used in 1,073 (64.7%) visits (Figure), with significant differences between racial and ethnic groups (P= 0.03). White patients were 26.8% more likely to receive abdominal CT and/or ultrasound than Black patients; 802 of 1,197 (67.0%) White patients received cross-sectional imaging compared to 124 of 234 (52.8%) Black patients (*P* = 0.01). Analyzed separately, White patients received more CT imaging alone than their Black counterparts (62.0% vs 49.6%), but the difference was not significant (*P* = 0.06). White patients also received significantly more ultrasound imaging than their Black counterparts (7.7% vs 5.8%, *P* = 0.02).

## DISCUSSION

In nationally representative ED data from 2013–2020, of over 12 million visits we found older Black patients with a chief complaint of abdominal pain were significantly less likely to receive definitive diagnostic imaging compared to White patients. We identified an absolute 14.2 percentage-point difference in cross-sectional imaging between Black and White patients. Our study builds on prior work demonstrating a racial disparity in abdominal imaging utilization in a single hospital’s ED for abdominal pain.^5^,^7^ It also adds to a greater body of evidence identifying racial disparities in imaging in the ED, specifically among older adults. There are no other national studies that focus on this specific age group with abdominal pain.^8^,^9^

The differential rate of imaging has at least two potential explanations. First, Black and White patients may present to the ED with a different distribution of underlying pathologies. While this cross-sectional study cannot exclude this possibility, for this to be the entire explanation, the demonstrated 26.8% greater imaging rate for White patients would need to be matched by a similarly elevated rate of severe, CT-diagnosable pathology among White compared to Black patients. The elevated rates of morbidity and mortality among Black older adults in US EDs and in admitted patients with emergency general surgical conditions belie this explanation.^17^,^18^ Second, White patients could be over-tested relative to their level of risk and, likewise, Black patients under-tested relative to their level of risk. In this case, the rates of CT utilization for Black patients described in this study could represent a clinically appropriate level of testing, or under-testing. Notably, in a single-ED study cohort that imaged at a similar rate to White patients in our data, two of five CTs obtained demonstrated acute findings.^7^ In either case, these differences in CT imaging reflect disparate levels of resource utilization, where greater resources in the form of CT scanner time are spent on White patients. To differentiate between these cases, a cohort that obtains imaging on all patients or follows patients past their discharge from the ED is required.

There is already a substantial body of literature that suggests the pain of Black patients in the ED may be minimized compared to the pain of White ED patients.^13^,^14^,^19^,^20^ These differences in the assessment of pain levels can also translate into disparate assessment and diagnosis. Therefore, given known disparities in the assessment and treatment of pain for Black patients, there may be disparities in imaging based on a chief complaint of abdominal pain.^11^ Biased cognitive patterns are particularly influential in abdominal pain because there are no objective signs, symptoms, standards, or lab tests that can perfectly determine the need for a CT, nor are there guidelines for the management of acute, geriatric abdominal pain.^6^,^11^,^21^ A cohort of ED patients at a single institution provides suggestive evidence of such an effect: Black and Hispanic adults (≥18) with abdominal pain were significantly less likely to receive CT than their White counterparts.^7^

Our work adds to the growing body of evidence highlighting disparate levels of testing for racially minoritized groups across various conditions. The actual mechanisms by which the health system produces biased care are multifactorial and require further attention.

## LIMITATIONS

This study has several limitations. It is possible that older Black patients are more likely to use the ED for lower acuity presentations, leading to appropriately lower rates of imaging. Similarly, we cannot rule out differential patient refusal of CT when offered by clinicians, for instance, due to differences in insurance or physician trust. Second, since the NHAMCS captures data from electronic health records, there is the possibility for level of measurement error based on self-reporting of race. The NHAMCS is also underpowered to assess differences in non-Black racial and ethnic groups. Additionally, the NHAMCS does not have the ability to ascertain clinician race/ethnicity, which may influence clinical bias. Third, the NHAMCS cannot differentiate between abdominal ultrasound and other ultrasounds. However, it is likely that most of these imaging modalities for a chief complaint of abdominal pain would be localized to the abdomen while a patient with a non-abdominal secondary reason for visit might receive an ultrasound, and any measurement error is unlikely to be systematic by race/ethnicity.

## CONCLUSION

This study revealed that Black older adults presenting to the ED with a chief complaint of abdominal pain receive significantly lower levels of cross-sectional imaging (CT and/or ultrasound) than White patients. Our findings highlight the need for further investigations into the causes of these disparities given the high rates of morbidity and mortality associated with abdominal pain ED presentations in older adults.

## Supplementary Information



## Figures and Tables

**Figure:**
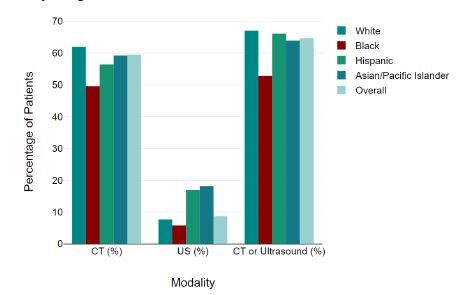
Diagnostic imaging by race and ethnicity. Percentages represent weighted percentages based on NHAMCS* national survey weights. *CT*, computed tomography *US*, ultrasound. (Source: *National Hospital Ambulatory Medical Care Survey, 2013–2020; authors’ calculations using nationally representative survey weights for percentages and total estimated emergency department visits)

**Table:** Patient demographic, geographic, and insurance characteristics; and hospital geography by race/ethnicity and overall.

Total estimated number of ED visits over 8-year period (Survey data for ED visits over 8-year period)	Patients, Number (%)					

	Total 12,553,136 (N=1,656)	White 9,028,252 (n=1,197)	Black 1,834,456 (n=234)	Hispanic 1,258,346 (n=153)	Other 432,082 (n=72)	P-value
Patient characteristics						
Age						<0.01
65–74 years	818 (49.7%)	563 (47.0)	141 (60.2)	93 (60.8)	21 (28.8)	
	[46.1–53.3%]	[43.4–50.8]	[50.8–68.9]	[51.0–69.8]	[18.5–41.8]	
75–84 years	556 (33.3)	414 (34.6)	64 (27.5)	42 (27.2)	36 (50.0)	
	[30.5–36.3]	[31.3–38.0]	[19.9–36.7]	[19.0–37.2]	[34.5–65.6]	
≥85 years	282 (17.0)	220 (18.4)	29 (12.3)	18 (12.0)	15 (21.2)	
	[14.0–20.4]	[15.1–22.2]	[8.2–18.1]	[6.8–20.6]	[9.3–41.5]	
Sex						0.19
Female	1,013 (61.1)	733 (61.2)	129 (55.3)	99 (64.9)	52 (72.8)	
	[57.7–64.4]	[57.3–64.9]	[47.0–63.2]	[53.6–74.7]	[58.8–83.5]	
Male	643 (38.9)	464 (38.8)	105 (44.7)	54 (35.1)	20 (27.2)	
	[35.6–42.3]	[35.1–42.7]	[36.8–53.0]	[25.3–46.5]	[16.6–41.2]	
Residence						0.60
Homeless	1 (0.1)	1 (0.1)	0 (0.1)	0 (0)	0 (0)	
	[0.0–0.3]	[0.0–0.5]	[0.0–1.2]	[0.0–0.0]	[0.0–0.0]	
Nursing	64 (3.8)	55 (4.6)	5 (2.2)	2 (1.0)	2 (3.1)	
	[2.6–5.4]	[3.02–6.8]	[0.6–7.6]	[0.3–4.3]	[0.8–11.4]	
Living at home/private	1529 (92.4)	1094 (91.4)	219 (93.7)	147 (96.1)	69 (95.4)	
	[89.9–94.3]	[88.0–94.0]	[88.5–96.6]	[90.3–98.5]	[86.7–98.5]	
Unspecified/other	62 (3.7)	47 (3.9)	10 (4.0)	4 (2.9)	1 (1.5)	
	[2.5–5.7]	[2.3–6.6]	[2.0–7.8]	[0.9–8.9]	[0.2–10.1]	
Insurance						<0.01
Medicaid/CHIP	89 (5.2)	34 (2.8)	22 (9.4)	19 (12.1)	14 (18.9)	
	[3.8–7.2]	[1.6–4.9]	[5.3–16.3]	[7.1–19.8]	[7.3–40.8]	
Medicare	1,322 (79.8)	991 (82.8)	173 (73.8)	109 (71.3)	49 (67.7)	
	[75.8–83.3]	[77.9–86.8]	[66.0–80.4]	[62.4–78.8]	[49.5–81.8]	
Private	109 (6.6)	71 (5.9)	18 (7.8)	15 (10.1)	5 (7.3)	
	[5.5–8.0]	[4.6–7.6]	[5.1–11.7]	[6.03–16.4]	[3.15–15.9]	
Self-Pay	16 (1.0)	14 (1.2)	0 (0.1)	2 (1.0)	0 (0.7)	
	[0.5–2.0]	[0.5–2.6]	[0.0–0.5]	[0.3–3.2]	[0.0–4.8]	
Unspecified/other	120 (7.4)	87 (7.3)	21 (8.9)	8 (5.5)	4 (5.4)	
	[5.0–10.7]	[4.5–11.9]	[5.02–15.3]	[2.4–12.6]	[2.1–13.2]	
Hospital geography						0.03
Urban	1,373 (82.9)	950 (79.4)	207 (88.6)	145 (94.9)	71 (98.5)	
	[72.5–90.0]	[67.0–88.0]	[69.7–96.4]	[85.8–98.3]	[89.9–99.8]	
Non-urban	283 (17.1)	247 (20.6)	27 (11.4)	8 (5.1)	1 (1.5)	
	[10.0–27.6]	[12.0–33.1]	[3.7–30.3]	[1.7–14.2]	[0.2–10.1]	
Triage level						0.16
Emergent	119 (7.2)	78 (6.5)	32 (13.5)	5 (3.0)	4 (5.8)	
	[5.4–9.5]	[5.0–8.7]	[8.6–20.7]	[0.7–11.2]	[2.1–15.0]	
Urgent	971 (58.6)	712 (59.5)	120 (51.2)	94 (61.8)	45 (62.0)	
	[53.4–63.6]	[53.7–65.0]	[42.4–60.0]	[47.8–74.0]	[43.7–77.4]	
Semi-urgent	105 (6.4)	72 (6.0)	16 (7.0)	14 (9.2)	3 (4.3)	
	[4.3–9.4]	[4.1–8.7]	[3.1–15.4]	[3.7–21.0]	[1.6–11.1]	
Non-urgent	18 (1.1)	12 (1.0)	5 (2.3)	0 (0)	1 (0.8)	
	[0.6–1.9]	[0.5–2.0]	[0.8–6.3]	[0.0–0.0]	[0.1–5.2]	
Unspecified/other	443 (26.7)	323 (27.0)	61 (26.0)	40 (26.0)	19 (27.1)	
	[21.6–32.6]	[21.5–33.2]	[17.4–37.0]	[17.0–37.9]	[14.1–45.9]	

(Source: NHAMCS, 2013–2020, authors’ calculations using nationally representative survey weights for percentages and total estimated ED visits).

*CHIP*, Children’s Health Insurance Program*; ED*, emergency department; *NHMACS*, National Hospital Medical Ambulatory Care Survey; *n*, number of observations in dataset before survey weighting.
