# Supplementary material for: Images in Black and White: Disparities in Utilization of Computed Tomography and Ultrasound for Older Adults with Abdominal Pain
Source: West J Emerg Med. 2025 Feb 28;26(3):452–7. doi: 10.5811/westjem.18087 (PMC12208078; doi:10.5811/westjem.18087)
Supplement: Supplementary file 1 [file wjem-26-452-s001.docx]

**Supplemental Table.** Reasons for visit Included in “abdominal pain” chief complaint.

| Reasons for visit | Number of observations N=1,042 | Percentage of observations |
| --- | --- | --- |
| Abdominal pain, cramps, spasms, NOS | 818 | 78.5 |
| Lower abdominal pain, cramps, spasms, NOS | 98 | 9.4 |
| Upper abdominal pain, cramps, spasms, NOS | 70 | 6.7 |
| Stomach and abdominal pain, cramps, spasm | 37 | 3.6 |
| Hernia of abdominal cavity | 10 | 1.0 |
| Abdominal distention, fullness, NOS | 5 | 0.5 |
| Bladder pain | 3 | 0.3 |
| Pain of liver, gallbladder, and biliary system | 1 | 0.1 |

(Source: Friedman et al 2021^5^)

*NOS*, not otherwise specified.
